# Superior Mesenteric Artery Syndrome: Clinical and Radiological Considerations

**DOI:** 10.1155/2015/628705

**Published:** 2015-08-12

**Authors:** M. Ezzedien Rabie, Olajide Ogunbiyi, Abdullah Saad Al Qahtani, Sherif B. M. Taha, Ahmad El Hadad, Ismail El Hakeem

**Affiliations:** ^1^Department of Surgery, Armed Forces Hospital, Southern Region, Khamis Mushait, Saudi Arabia; ^2^Department of Radiology, Armed Forces Hospital, Southern Region, Khamis Mushait, Saudi Arabia

## Abstract

*Background*. Superior mesenteric artery (SMA) syndrome is a rare condition of duodenal obstruction, caused by the overlying SMA. *Aim*. To report on our experience with the management of SMA syndrome, drawing the attention to its existence. *Material and Methods*. We reviewed our records to identify cases diagnosed with SMA syndrome, in the period from October 1995 to January 2012. *Results*. Seven patients were identified, one male and six females. Their mean age was 17.1 years. Vomiting and abdominal pain were the presenting complaints in all patients and history of weight loss was present in six of them. In no patient was the diagnosis suspected initially on clinical grounds. Only after radiological investigations was the diagnosis declared. Radiology took the form of gastrografin/barium meal only in four patients and both gastrografin/barium meal and computerized tomography scan in the remaining three. Four patients responded to medical treatment and surgery was performed in the remaining three, with open duodenojejunostomy in two patients and laparoscopic dissection of the ligament of Treitz in the third. Long lasting improvement was sustained in all patients except one in the surgery group who, despite initial improvement, still has infrequent attacks of abdominal pain. *Conclusion*. Although the clinical manifestations of SMA syndrome are shared with many other disease entities, it has unique radiological as well as endoscopic features, which enables a confident diagnosis to be made. Once diagnosed, conservative treatment with nutritional support and positioning should be tried first. In case of unresponsiveness, surgery may give a lasting cure.

## 1. Introduction

Superior mesenteric artery (SMA) syndrome was first described by Professor Rokitansky in 1842 [1, 2]. Only relatively recently has it established itself as a disease entity. The clinical picture is caused by compression of the 3rd part of the duodenum between the SMA and aorta, which exert a clam-like or striding action on it. Virtually, any condition associated with weight reduction may be followed by the syndrome. Tuberculosis [3], brucellosis [2], diabetes mellitus [4], anorexia nervosa [5], blunt abdominal trauma [6], and burns [7] are only few to mention. It has also been reported after spinal surgery [8], application of body casts [9], and bed confinement in the supine position [10]. Moreover, it may complicate weight reduction following bariatric surgery [11], a pertinent fact to consider after the current surge of this type of surgery.

## 2. Material and Methods

We reviewed our records to identify cases of SMA syndrome, diagnosed in our hospital, a 609-bed tertiary referral hospital, serving a population of 1 million individuals, in the period from October 1995 to January 2012. Patients' files were retrieved and data were collected which included patients' demographics, their clinical presentation, how the diagnosis was substantiated, the treatment offered, and the response to treatment.

## 3. Results

In this period, we were able to retrieve the files of seven patients, one male and six females, with a mean age of 17.1 years (range 9–25, SD 5.3).

All patients had vomiting and all of them had abdominal pain which was acute in three cases and chronic in the other four. Weight loss was spontaneous in five patients and it followed a weight reduction program in one, while in the seventh patient no history of weight loss was obtained. Associated comorbidities were present in two patients, one with tuberculous interstitial nephritis and another with traumatic paraplegia, while the rest had no comorbidities.

Out of the 7 patients, only three underwent upper endoscopy and in none of them was it diagnostic.

**Figure 1:**
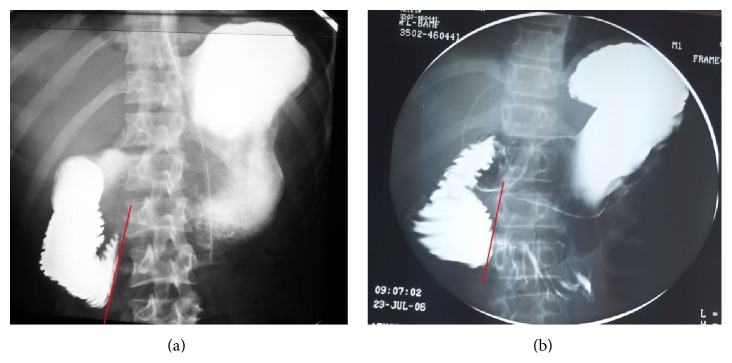
(a) and (b) Dilation of the duodenum with abrupt cut-off at its third part, coinciding with the line of the SMA (red line).

**Figure 2:**
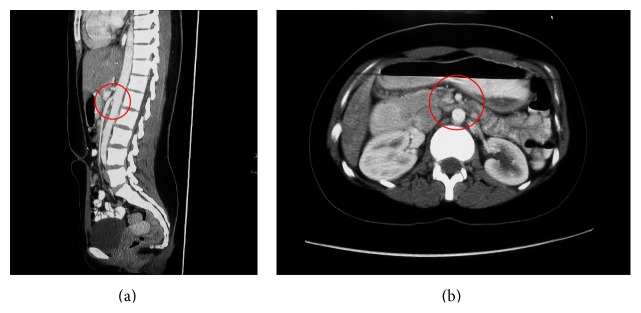
Narrow aortomesenteric angle (10°) and aortomesenteric distance (6 mm) (red circles in (a) and (b), resp.), compressing the duodenum in between.

The diagnosis was not suspected on clinical grounds. Rather, it was revealed after radiological investigations performed to explore the patients' complaints. This took the form of gastrografin/barium meal only in four patients (Figure 1), while both gastrografin/barium meal and computerized tomography (CT) scan were used in the remaining three (Figure 2).

Four patients responded to medical treatment, the essential elements of which were initial gastroduodenal decompression through a nasogastric tube, followed by nutritional support with total parenteral nutrition (TPN) or, if tolerated, small frequent oral meals, aided by positioning of the patient in the right recumbent or prone positions to relieve the compressed duodenum. In the remaining three, surgery was performed. In two patients, it was the primary treatment in the form of side to side duodenojejunostomy, while in the third patient, it followed unresponsiveness to medical treatment and took the form of laparoscopic dissection of the ligament of Treitz.

All patients improved with no further admissions with the same complaints except one patient who received primary duodenojejunostomy, in whom vomiting recurred and was admitted several times with left iliac fossa pain with no obvious reason despite repeated investigations, including a psychiatric evaluation. The clinical and radiological features of the patients as well as the treatment given are shown in Tables 1 and 2.

## 4. Discussions

Wilkie described the clinical and pathophysiological characteristics of the syndrome as well as its management approach, in a series of 64 patients [12, 13], giving the syndrome its eponym “Wilkie's syndrome.” Many other eponyms, including chronic duodenal ileus, megaduodenum, aortomesenteric artery compression, arteriomesenteric duodenal obstruction, cast syndrome, and chronic duodenal pseudoobstruction, have also been used [10, 14]. The diagnosis requires a high index of suspicion in the proper clinical context and entails a detailed radiologic evaluation.

Clinically, the patient with a predisposing illness presents with features of gastric outlet obstruction. Sense of fullness, postprandial epigastric pain, belching, and vomiting are characteristic features. Radiologically, barium meal and CT scan show dilatation of the stomach and proximal duodenum with an abrupt cut-off across its third part, together with a decreased aortomesenteric distance as well as aortomesenteric angle. These findings, in the proper clinical sitting, virtually establish the diagnosis. Ultrasound (US) has also been used to aid in the diagnosis. The findings include to and fro movements across the duodenum in the supine, left recumbent, and sitting positions, with facilitation of the flow through the jejunum and elongation of the aortomesenteric distance when the patient assumes the right recumbent position [15]. These findings confirm the diagnosis and establish the role of positioning in providing a symptomatic relief in such cases. Despite its confirmatory role, US was not used in any of our patients to substantiate the diagnosis, probably due to its inability to provide clear anatomic details, compared to barium meal or CT scan.

**Figure 3:**
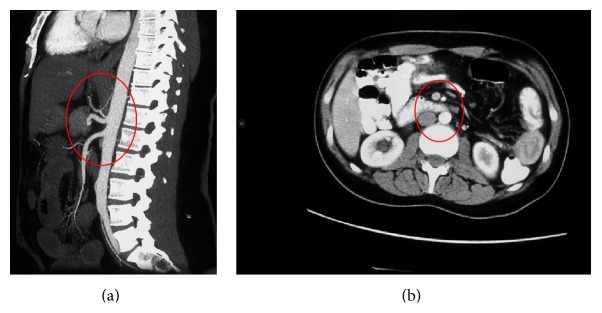
(a) and (b) Wide aortomesenteric angle and distance in a normal individual with no duodenal compression (red circles).

The syndrome has specific anatomic basis. The SMA takes off from the abdominal aorta at the level of the first lumbar vertebra with an average angle of 42.4° (range 18° to 70°) and a distance of 10–28 mm. Suspended by the ligament of Treitz, which is attached to its 4th part or to its junction with the jejunum, the duodenum crosses the abdomen at the level of the third lumbar vertebra [10]. Minor anatomic alterations predispose to the clinical manifestations of the syndrome. A narrow aortomesenteric angle of 15.2° (range 1°–40°) and a narrow aortomesenteric distance of 2 to 8 mm have been observed in individuals with SMA syndrome [10]. As seen in Figures 2 and 3, CT in a formatted sagittal view could easily document these measures in individuals with clinical evidence of the syndrome, thus establishing the diagnosis.

Thinning out of the fat pad between SMA and aorta, consequently upon weight loss, narrows the aortomesenteric angle and distance, thereby compressing the duodenum and thus producing the clinical manifestations of the syndrome. Other contributory factors include an abnormally low origin of SMA, excessive lumbar lordosis, and hypertrophied or shortened ligament of Treitz or its multiple attachments to the duodenum [10]. High fixation of the duodenum by the ligament of Treitz or an anomalous SMA crossing directly over the aorta as the latter transects the duodenum [16] has also been incriminated.

When performed, endoscopy may reveal narrowing of the 3rd part of the duodenum due to external compression [2]. This was not noticed in our series where upper endoscopy was performed in only three cases, probably due to the unfamiliarity of the endoscopist with the condition or the inability to reach the 3rd part of the duodenum.

As noticed in our series, upper gastrointestinal series utilizing barium or gastrografin will show dilatation of the stomach and duodenum down to the 3rd part, with a sudden cut-off distally, conforming to the anatomical position of the superior mesenteric artery. In addition, CT scan will show the anatomical configuration of the region with the clam-like action of the SMA and aorta across the third part of the duodenum, resulting in the abrupt cut-off. On sagittal reconstruction of the CT images, a narrowed aortomesenteric distance and angle can be easily depicted, substantiating the diagnosis.

Unrelieved, duodenal perforation may ensue [17]. For established cases of SMA syndrome, medical and surgical options do exist. It is intuitive to start with the medical lines first which include decompression of the stomach and duodenum with a nasogastric tube, correction of nutritional and electrolytes deficiencies, through TPN [2], or preferably, if possible, enteral feeding with a nasojejunal tube past the point of compression, which facilitates the nutritional management while avoiding TPN complications. When tolerated, oral feeding may be resumed. This helps build up the fat cushion between the SMA and aorta and, hence, reversing the situation. Additionally, as it lies in the root of the mesentery, SMA may be dragged by the small bowel, to drop off the duodenum when the patient assumes the prone or right recumbent position, as proved by ultrasound studies [15]. This might bring about symptomatic relief till the fat pad builds up.

Failing appropriate medical treatment, surgical intervention may be considered. The essence is to bypass the site of obstruction by anastomosing the bowel below the bowel above it, thus resuming the functional integrity of the bowel. This may take the form of gastrojejunostomy or duodenojejunostomy, by the open [18] or laparoscopic means [19]. Dissection of the ligament of Treitz, with mobilization of the 3rd and 4th parts of the duodenum, releasing the compression, has also been reported [20, 21]. More recently, robotic duodenojejunostomy has been utilized with success [22]. In our series, four patients successfully responded to conservative treatment, one patient failed to respond and received laparoscopic dissection of the ligament of Treitz, and the remaining two received duodenojejunostomy without a proper trial of medical treatment. Although both improved postoperatively, one of them has had repeated emergency room visits for left iliac fossa pain with no obvious reason. In this regard, surgery should not be offered before a proper trial of conservative management.

Other pathological conditions with similar clinical presentation, including diabetic gastroparesis, scleroderma with duodenal involvement [23], hereditary megaduodenum [24], megaduodenum due to aganglionosis [25], have been rarely reported. The distinction between these entities and SMA syndrome is of utmost importance when embarking on treatment, especially the surgical option.

The apparent rarity of SMA syndrome may reflect its true rare nature or, alternatively, unawareness of its existence. Published articles are mainly case reports and, rarely, small case series. This limits our understanding of the disease. Keeping a high index of suspension, followed by the utilization of appropriate radiology, may bring more cases to light.

## 5. Conclusion

SMA syndrome is a rarely diagnosed condition. Keeping a high index of suspicion followed by the utilization of appropriate radiology is essential for its diagnosis. Although the clinical manifestations of SMA syndrome are shared with many other disease entities, it has unique radiological as well as endoscopic features, which enable a confident diagnosis to be made. Once diagnosed, conservative treatment with nutritional support and positioning should be tried first. In case of unresponsiveness, surgery may give a lasting cure.

## Figures and Tables

**Table 1:** Clinical, radiological, and endoscopic features of individual patients in the series.

Patient	Age (years)/ sex	Clinical presentation	Diagnosis on gastrografin/ barium meal^*∗*^	Diagnosis on CT scan^*∗∗*^	Upper endoscopy
1st	17/♀	Chronic abdominal pain, vomiting, and weight loss	Yes	Not done	Done, not diagnostic

2nd	16/♀	Chronic abdominal pain, nausea, repeated vomiting, persistent hunger, and weight loss	Yes	Yes	Done, not diagnostic

3rd	18/♀	Acute abdominal pain, vomiting, and weight loss	Yes	Not done	Not done

4th	25/♂	Acute abdominal pain, vomiting, sense of distension, and weight loss	Yes	Yes	Done, not diagnostic+

5th	13/♀	Chronic abdominal pain after meals, vomiting, and weight loss	Yes	Not done	Not done

6th	9/♀	Acute abdominal pain, nausea, and repeated vomiting	Yes	Not done	Not done

7th	22/♀	Chronic abdominal pain, vomiting, and weight loss	Yes	Yes	Not done

^*∗*^Dilation of the duodenum with abrupt cut-off at its third part, coinciding with the line of the SMA.

^*∗∗*^Narrow aortomesenteric angle and aortomesenteric distance.

**Table 2:** Treatment and its result.

Patient	Treatment	Result
1st	Duodenojejunostomy	Recurrence of vomiting
2nd	Medical treatment	Improved
3rd	Duodenojejunostomy	Improved
4th	Medical treatment	Improved
5th	Laparoscopic dissection of the ligament of Treitz	Improved
6th	Medical treatment	Improved
7th	Medical treatment	Improved
